# Management of Long QT Syndrome: A Systematic Review

**DOI:** 10.7759/cureus.62592

**Published:** 2024-06-18

**Authors:** Wilhelmina N Hauwanga, Ryan Chun Chien Yau, Kang Suen Goh, Jose Ittay Castro Ceron, Berley Alphonse, Gurinder Singh, Sara Elamin, Vaishnavi Jamched, Aaron A Abraham, Joshi Purvil, Jeshua N Devan, Gabriella Valentim, Billy McBenedict, Bruno Lima Pessôa, Evandro T Mesquita

**Affiliations:** 1 Family Medicine, Faculty of Medicine, Federal University of the State of Rio de Janeiro, Rio de Janeiro, BRA; 2 Internal Medicine, Monash University Malaysia, Johor Bahru, MYS; 3 Neurosurgery, Fluminense Federal University, Niterói, BRA; 4 Public Health, Fluminense Federal University, Niterói, BRA; 5 Clinical Medicine, Antônio Pedro University Hospital, Niterói, BRA

**Keywords:** left cardiac sympathetic denervation (lcsd), therapeutic, beta-blockers, implantable cardioverter-defibrillator (icd), long qt

## Abstract

Long QT syndrome (LQTS) is a cardiac disorder characterized by prolonged repolarization of the heart's electrical cycle, which can be observed as an extended QT interval on an electrocardiogram (ECG). The safe and effective management of LQTS often necessitates a multifaceted approach encompassing pharmacological treatment, lifestyle modifications, and, in high-risk cases, the implantation of implantable cardioverter-defibrillators (ICDs). Beta-blockers, particularly nadolol and propranolol, are foundational in treating LQTS, especially for high-risk patients, though ICDs are recommended for those with a history of cardiac arrest or recurrent arrhythmic episodes. Intermediate and low-risk patients are usually managed with medical therapy and regular monitoring. Lifestyle modifications, such as avoiding strenuous physical activities and certain medications, play a critical role. Additionally, psychological support is essential due to the anxiety and depression associated with LQTS. Left cardiac sympathetic denervation (LCSD) offers an alternative for those intolerant to beta-blockers or ICDs. For diagnosis and management, advancements in artificial intelligence (AI) are proving beneficial, enhancing early detection and risk stratification. Despite these developments, significant gaps in understanding the pathophysiology and optimal management strategies for LQTS remain. Future research should focus on refining risk stratification, developing new therapeutic approaches, and generating robust data to guide treatment decisions, ultimately aiming for a personalized medicine approach.

## Introduction and background

The heart functions as a precise electromechanical pump, its rhythmic beating orchestrated by the electrical impulses coursing through cardiomyocytes [1]. This process relies on the sequential generation and propagation of action potentials, which regulate the contraction and relaxation phases of the cardiac cycle [2]. During depolarization, sodium and calcium ions influx inwardly, initiating cellular excitation, while potassium effluxes outwardly, facilitating repolarization and preparing the cell for subsequent contractions [2]. Any disruption in this finely tuned electrical activity, whether in the form of altered ion currents or irregular action potential propagation, can precipitate severe arrhythmias [3]. These deviations in action potential propagation are often rooted in genetic variations affecting the proteins responsible for cardiac ion channels, disrupting the delicate balance of ion currents and compromising the heart's electrical stability [4].

In individuals over 40 years old, ischemic heart disease ranks as the primary cause of sudden cardiac death (SCD), whereas in younger populations (<35 years old), arrhythmic syndromes, including cardiomyopathies and cardiac channelopathies, predominate [4]. Notably, recent studies indicate that approximately 30% of negative autopsies in individuals under 15 years old could be attributed to channelopathy-related gene variations [5]. Among the main cardiac channelopathies are Brugada syndrome, long QT syndrome (LQTS), short QT syndrome, and catecholaminergic polymorphic ventricular tachycardia, each characterized by distinct pathogenic genetic variations affecting essential cardiac channels [4].

LQTS stands out as the most prevalent cardiac channelopathy, with an estimated occurrence rate of one in 2,500 individuals [6]. Notably, LQT2 emerges as a significant risk factor for patients within the first nine months postpartum to experience a cardiac event [7]. The mortality rate among symptomatic LQTS patients left untreated is notably high, with approximately 21% experiencing mortality within one year from the first syncope episode. Ventricular tachyarrhythmia, predominantly TdP, underpins the cardiac events in LQTS, posing a grave risk of cardiac arrest or sudden death [8].

Pharmacological agents are a common cause of QT prolongation. Polypharmacy may lead to fetal QT prolongation, due to augmented QT prolongation. Discontinuing the offending drug often normalizes the QT interval. Medications that can prolong the QT interval include (1) antipsychotics: haloperidol, ziprasidone, quetiapine, thioridazine, olanzapine, risperidone, and droperidol; (2) antiarrhythmics: amiodarone, sotalol, dofetilide, procainamide, quinidine, and flecainide; (3) antibiotics: macrolides and fluoroquinolones; and (4) antidepressants: amitriptyline, imipramine, citalopram, and amitriptyline [9]. Caution is advised when combining QT-prolonging medications or using them in patients with electrolyte abnormalities, such as hypokalemia. 

Congenital LQTS is primarily caused by mutations in genes encoding cardiac ion channels, leading to prolonged action potentials and increased risk of arrhythmias. The most common forms, type 1-3 long QT syndrome (LQT1, LQT2, and LQT3), account for 75% of cases and involve mutations in the KCNQ1, KCNH2, and SCN5A genes, respectively. These mutations disrupt the potassium and sodium currents critical for heart repolarization, causing prolonged QT intervals. Genetic testing has identified over 300 mutations, predominantly missense, affecting various ion channels. In LQT1, the loss-of-function mutation in KCNQ1 affects the slowly activating delayed rectifier potassium current (IKs current), preventing the corrected QT interval from appropriately shortening during exercise, aiding in the diagnosis. Other LQTS types, such as LQT4 through LQT17, involve mutations in additional genes, highlighting the syndrome's genetic heterogeneity [10].

The diagnosis of LQTS entails a comprehensive evaluation comprising a detailed family history and electrocardiogram (ECG) assessment. LQTS's key indicator symptoms include syncope, seizures, or palpitations [11]. Additional investigations, including exercise stress tests and Holter monitoring, may be warranted to assess cardiac function and identify arrhythmic events [11]. In addition, provocative tests utilizing drug challenges can aid in distinguishing LQTS from other types of arrhythmias, enhancing diagnostic accuracy [11]. Management of LQTS involves a multidisciplinary approach aimed at reducing the risk of life-threatening arrhythmias and SCD. Understanding the intricacies of LQTS is crucial for effective management and improved outcomes in affected individuals. This paper aims to delve into the nuances of LQTS for a comprehensive understanding of the management and treatment modalities.

## Review

Materials and methods

The systematic review adhered to the principles outlined in the Preferred Reporting Items for Systematic Reviews and Meta-Analyses (PRISMA) guidelines for the organization and reporting of its results.

Source Information and Search Strategy

An electronic search was performed across multiple research databases, including PubMed, Embase, Scopus, and Web of Science (Table 1). PubMed was accessed on January 12, 2024; Embase on January 13, 2024; and Scopus and Web of Science on January 14, 2024. The search spanned the period between January 2018 and March 2024. For Web of Science, fields that were not related to medicine or cardiology were excluded (e.g. physics fluids plasmas or medical ethics).

**Table 1:** Summary of the search strategy from the databases

Database	Search strategy (long QT syndrome)	Filters used
PubMed	((Treatment[Title/Abstract] OR Therapeutics[Title/Abstract] OR therapy[Title/Abstract])) AND ((“long qt syndrome”[Title/Abstract] OR "romano ward syndrome”[Title/Abstract] OR “timothy syndrome”[Title/Abstract] OR “andersen syndrome”[Title/Abstract]))	Humans only, English language, exclude preprints, filter years 2018 - 2024
Embase	(treatment:ab,ti OR therapeutics:ab,ti OR therapy:ab,ti) AND ('long qt syndrome':ab,ti OR 'romano ward syndrome':ab,ti OR 'timothy syndrome':ab,ti OR 'andersen syndrome':ab,ti) AND ([controlled clinical trial]/lim OR [randomized controlled trial]/lim) AND [humans]/lim AND [english]/lim AND [embase]	Humans only, English language, exclude preprints, filter years 2018 - 2024
Scopus	((treatment (Article title, Abstract, Keywords) OR therapeutic (Article title, Abstract, Keywords) OR therapy (Article title, Abstract, Keywords)) AND (( 'long AND qt AND syndrome' OR 'romano AND ward AND syndrome (Article title, Abstract, Keywords)' OR 'timothy (Article title, Abstract, Keywords) AND syndrome' OR 'andersen AND syndrome (Article title, Abstract, Keywords))	Humans only, English language, exclude preprints, filter years 2018 - 2024
Web of Science	(treatment OR therapeutics OR therapy) (Abstract) AND ('long qt syndrome': OR 'romano ward syndrome' OR 'timothy syndrome' OR 'andersen syndrome') (Abstract)	Humans only, English language, exclude preprints, filter years 2018 - 2024

Inclusion and Exclusion Criteria

The inclusion criteria encompassed studies involving human subjects of any age diagnosed with LQTS and undergoing treatment. Eligible study designs included primary research studies published in English. Clinical cases were included only if they involved more than 10 participants. Studies reporting on the treatment and management of patients with LQTS were of interest. Only peer-reviewed journal articles in English were considered for inclusion. Exclusion criteria encompassed non-human or in vitro studies that did not directly inform on LQTS and their management, non-peer-reviewed articles, conference abstracts, and editorials.

Results

**Figure 1:**
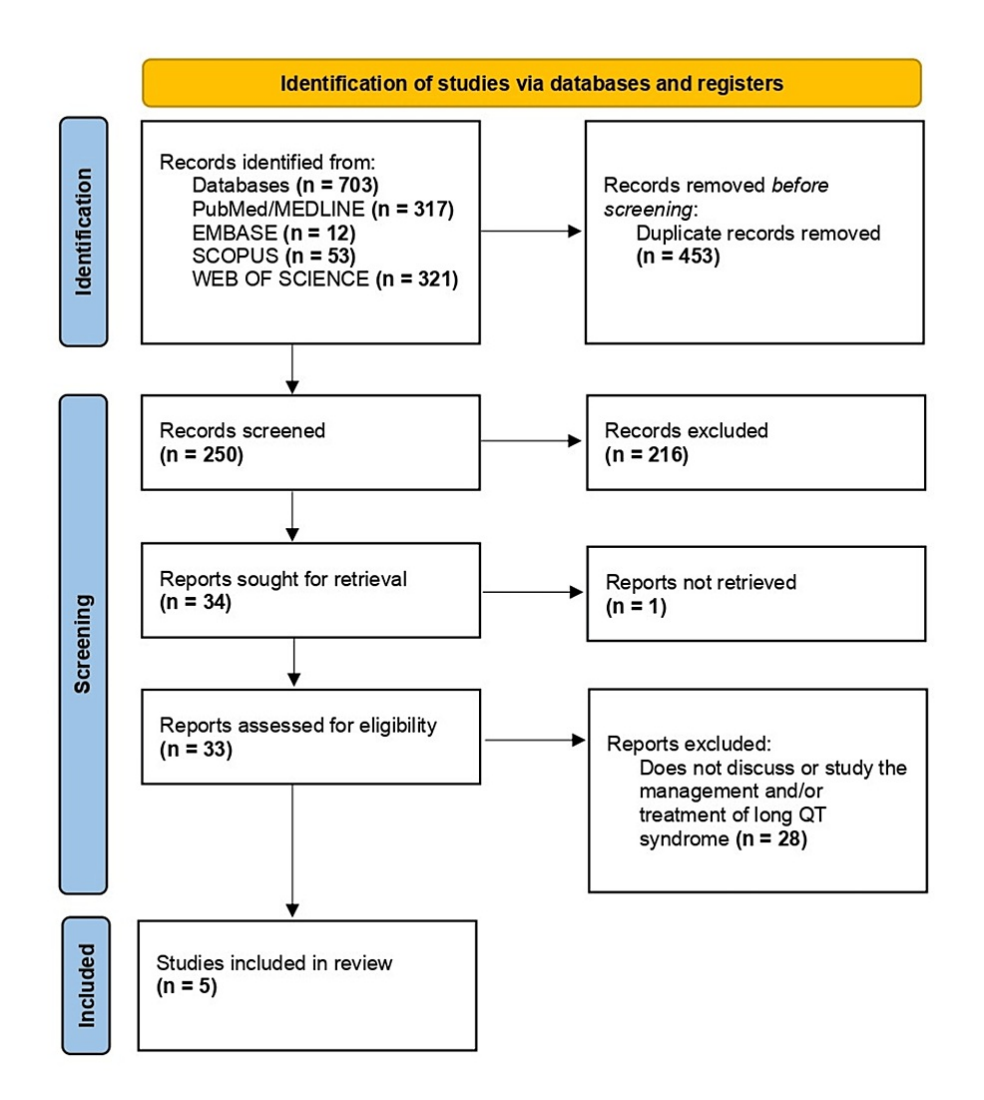
Preferred Reporting Items for Systematic Reviews and Meta-Analyses (PRISMA) flow diagram indicating the steps taken to filter the articles for this review

Through our search strategy, we identified a total of 703 articles (Figure 1), comprising 317 from PubMed/Medline, 12 from Embase, 53 from Scopus, and 321 from Web of Science. Filters were applied based on the inclusion/exclusion criteria. The articles were transferred to an Excel sheet, where 453 duplicates were manually removed, resulting in 250 articles. These 250 articles were further scrutinized based on their titles and abstracts, leading to the disqualification of 216, leaving 34 articles. Full texts for one article could not be retrieved, leaving us with 33 papers for eligibility assessment. After a thorough full-text review, 28 papers were excluded, resulting in five articles being included in the final review (Table 2). Data screening was independently conducted by two review authors, with a third reviewer consulted in cases of disagreement. Notably, no automated tools were utilized in this process.

**Table 2:** Studies that were used to synthesize this review, with their respective demographics and key results LCSD = Left Cardiac Sympathetic Denervation; IDC = Implantable Cardioverter-Defibrillator; LQTS = Long QT Syndrome; cLQTS = Congenital Long QT Syndrome; QTc = Corrected QT; TWA = T-Wave Alternans; TdP = Torsades de Pointes; SCD = Sudden Cardiac Death

Author	Participants (n, age range, sex)	Key findings
Ahn et al. [12]	105 patients: 48 women and 57 men	β-blockers are used as the first-line treatment. mexiletine is recommended for LQT3. Normal potassium levels are essential for LQT2 and LQT7, which may require supplementation. For LQT1 patients, if syncope occurs despite treatment, LCSD is considered before implanting an ICD, especially in children. The ICD placement is indicated in cases/history of cardiac arrest. The study found that individuals with LQT3 experienced more cardiac arrests compared to those with LQT1. Consequently, a significantly higher number of LQT3 patients underwent ICD insertion compared to other LQTS groups, suggesting a greater likelihood of ICD implantation for LQT3 patients than those with other genotypes.
Krøll et al. [13]	500 patients (192 subjects with β-blocker treatment break; 308 subjects with no β-blocker treatment break) - median age is 35 y/o - the interquartile range is 17–50 y/o - 185 males, 315 females	Among the 500 patients who initiated β-blocker therapy subsequent to their cLQTS diagnosis, the most commonly prescribed β-blockers were metoprolol (72.4%), followed by atenolol (5.8%), bisoprolol (3.4%), propranolol (2.0%), carvedilol (1.0%), and nebivolol. It is currently recommended (Class Ib) that cLQTS patients who receive an ICD for secondary prophylaxis should also undergo concurrent treatment with β-blockers post-ICD implantation.
Niaz et al. [14]	204 subjects (140 had LCSD+pharmacotherapy; 64 had LCSD monotherapy). Participants’ current age: N/A - participants’ age at diagnosis was 13.2±12.6 years - 88 males and 116 females	LCSD can serve as a safe and efficacious stand-alone treatment in a meticulously chosen subset of individuals with low-risk LQTS, particularly those experiencing intolerable side effects from beta-blocker medication. Patients opting for LCSD monotherapy typically exhibited low-risk profiles, characterized by a median QTc interval below 500 ms, infrequent symptoms, and a minimal occurrence of cardiac events before LCSD while on pharmacotherapy.
Takasugi et al. [15]	n = 11, age range = 17.36 (0–63); sex = female 4, male 7	Beta-blocker therapy significantly diminishes both the peak level and occurrence frequency of TWA, alongside a reduction in syncope and TdP episodes. Beta-blockers exert their effect by not only lowering baseline heart rate but also mitigating sudden increases in heart rate, thus suppressing both the magnitude and frequency of TWA.
Wang et al. [16]	Overall: 3035, non-ICD: 2438, ICD: 597, ICD patient: male: 185 (31.0%), female:412 (69%), age: 24 (14–38)	ICD therapy demonstrated a reduced risk of all-cause mortality, all-cause mortality before the age of 50, and SCD in the LQTS population. Additionally, it was associated with a decreased risk of all-cause mortality across various indication subgroups. These findings offer compelling evidence for the implementation of ICD implantation in high-risk LQTS patients.

Analysis of Study Quality/Bias

The quality of the six articles was assessed using the JBI Critical Appraisal Tools (Appendix Table 3). The JBI appraisal tool includes questions that allow for the assessment of the quality of articles in a systematic review. This is vital as it ensures the reliability and validity of our findings by critically evaluating each study's methodological rigor. It also allows for the identification of biases, errors, or flaws that may compromise the accuracy of the conclusions drawn from the synthesized evidence. This process also led to the removal of poor-quality articles. All studies included in the analysis focused on a clearly defined issue regarding the management of LQTS.

Each study recruited participants in an acceptable manner, clearly stating inclusion criteria and participants' demographic data (Appendix Table 3). However, numerous studies displayed significant differences in gender representation among participants, with a particularly low proportion of male participants compared to females. This imbalance presents difficulties in making meaningful comparisons.

Discussion 

To date, the safe and effective management of patients with LQTS often necessitates the implantation of an ICD. While ICD therapy is currently recommended for primary prevention of sudden death, its placement may not always be practical or technically feasible. High-risk LQTS patients and those who have experienced cardiac arrest or multiple arrhythmic syncopal episodes should undergo aggressive management with ICD placement, often alongside medical therapy (beta-blockers) to mitigate future episodes, though there are risks of inappropriate treatment due to oversensing of T-waves. Intermediate or low-risk LQTS patients are frequently managed with medical therapy and longitudinally observed for persistent symptoms [17].

For individuals diagnosed with LQTS, the management strategy includes a focus on preventing lethal cardiac arrhythmias such as ventricular tachycardia/ventricular fibrillation. Treatment options range from pharmacological interventions to surgical and device-based solutions, tailored according to the patient's symptoms and risk levels identified through risk stratification. High-risk factors include female gender, LQT2 genotype, and corrected QT (QTc) prolongation ≥500 milliseconds. Additionally, psychological support may be necessary due to heightened anxiety and depression linked to the condition and its treatments, particularly ICDs, necessitating involvement from trained psychologists beyond genetic counselors and clinicians. It is also vital to screen family members and first-degree relatives, as they might also need treatment due to common mutations in LQTS1 and LQTS2 genes [18].

Lifestyle Modification

Lifestyle modification remains a crucial complement to medical, surgical, or device therapy in managing LQTS. This entails avoiding extreme physical exertion, such as swimming and diving, as well as minimizing exposure to auditory stimuli such as alarm clocks, and being cautious with a wide array of medications listed on QTdrugs.org, to prevent recurrent symptoms [19].

Pharmacological Treatment 

The management of LQTS emphasizes the use of beta-blockers as the primary treatment option. For patients with LQT3, sodium channel blockers such as flecainide and mexiletine are recommended as adjunct therapies to potentially shorten the QT interval, with mexiletine considered a secondary treatment option. In cases of recurrent syncope and challenges in medical management, the implantation of an implantable ICD, alongside beta-blocker therapy and atrial pacing, may be necessary. Treatment strategies tailored to specific genotypes, such as adding mexiletine for LQT3 and left cardiac sympathetic denervation (LCSD) for LQT1, are advised to align with genotype-phenotype correlations [12,15].

β-Blockers

Medical therapy for LQTS, particularly types LQT1 and LQT2, relies heavily on high-dose β-blocker treatment, such as nadolol or propranolol, administered at 2-4 mg/kg/day. β-blockers are established as the cornerstone of treatment for these subtypes, effectively reducing syncope and SCD risks. A study showed that β-blockers significantly shorten the QTc interval and could be potentially useful for monitoring medication adherence in LQTS patients through routine ECGs [20].

Propranolol, known for its sodium channel-blocking properties independent of β-adrenergic pathways, proves especially beneficial for patients with LQT1 and LQT2 who experience cardiac events under adrenergic stress such as physical or emotional exertion. Its effectiveness is further affirmed by its role in ameliorating arrhythmia phenotypes in KCNQ1-mutant myocardial models, indicating a substantial therapeutic benefit across various mechanisms [21,22]. β-blockers are recommended universally for managing congenital long QT syndrome (cLQTS), classified as class I therapy for symptomatic cases and class II for asymptomatic carriers with a normal QT interval. This treatment significantly decreases the incidence of malignant ventricular arrhythmias (VAs) and SCD, life-saving intervention for high-risk cLQTS patients [13,23].

The Role of ICD 

The role of ICD extends beyond the reduction of T-wave alternans (TWA) and peak amplitude, correlating with fewer instances of syncope and TdP. This variability suggests that the effectiveness of β-blockade may differ based on the heart rate dependency of the TWA, with some LQTS patients benefiting more than others [12,15]. Furthermore, propranolol and nadolol surpass other β-blockers such as metoprolol in preventing cardiac events, with particular efficacy in reducing cardiac repolarization time in patients with markedly prolonged QTc intervals, providing crucial insights for tailored LQTS management [12].

LCSD

Left cardiac sympathetic denervation has become an important treatment strategy for preventing SCD in LQTS, particularly before considering an implantable ICD in younger patients. The antifibrillatory effects of LCSD, such as attenuating localized sympathetic discharge and increasing the ventricular fibrillation threshold, have been documented to significantly reduce cardiac events by about 90% after the procedure. This makes LCSD a valuable option for managing patients with LQTS, especially in children or in cases of breakthrough syncope in LQT2 and LQT3, where there is a high susceptibility to sympathetic surges [12,15].

While LCSD is effective, it is not considered curative and should be selected for patients after careful risk assessment. It serves as a safe and potentially standalone therapy for certain low-risk patients who experience adverse effects from β-blockers, significantly improving their quality of life without the long-term commitments or complications associated with ICDs [14].

ICD 

Implantable ICDs are important in managing LQTS, particularly for patients with LQT3, who show reduced responsiveness to β-blockers and often experience cardiac events during rest or sleep. These patients, exhibiting a higher risk for SCD due to arrhythmias such as atrioventricular block and sinus arrest, frequently benefit from ICDs as primary treatment, especially when β-blockers fail to prevent syncope or ventricular tachycardia. Guidelines suggest ICD placement as a class II indication for LQTS patients experiencing life-threatening arrhythmias despite β-blocker therapy, highlighting its importance in preventing sudden death among high-risk groups [24].

The decision to implement ICD therapy must be carefully considered, especially given the device's association with complications such as inappropriate shocks and the psychosocial impact on quality of life. For LQT3 and other rarer forms of LQTS, where genetic and clinical data are limited, the management often includes avoiding QT-prolonging medications and careful monitoring of physical activity levels. Despite these challenges, ICD therapy has demonstrated a significant reduction in all-cause mortality and SCD in the LQTS population, supporting its use in specific patient subgroups defined by severe clinical manifestations and genetic predispositions [13,23].

Furthermore, an interdisciplinary approach involving cardiogenetics plays a crucial role in optimizing the management of LQTS, enabling a nuanced understanding of each patient’s risk and tailoring ICD use accordingly. While ICDs significantly enhance survival rates, ongoing research and refined risk stratification are essential to ensure appropriate application and avoid unnecessary procedures in lower-risk patients. This is particularly crucial as the landscape of LQTS management continues to evolve with advances in genetic insights and therapeutic technologies [16,25].

Vitamin/Supplements and Other Medications

Magnesium supplementation has been shown to enhance potassium ion transport, stabilize cell membranes, and improve ECG stability, making it a vital component in the management of LQTS. Research has demonstrated significant reductions in field potential duration (FPD) across various models, underscoring the broad mechanisms by which magnesium supports cardiac function and rhythm stability [21,22].

Potassium supplementation has also been beneficial, particularly in preventing recurrent syncope in LQTS2 patients. It works by enhancing the repolarizing potassium current (IKr), which is inversely regulated by extracellular potassium levels, thereby contributing to a more stable cardiac repolarization process and reducing arrhythmic risks [19]. A study reported [26] the use of injectable calcium gluconate (900 mg over 24 hours in 5% dextrose) to be effective, with a normalization of the QTc interval observed after 24 hours of infusion. The treatment was continued until the serum calcium level stabilized within the normal range, leading to the cessation of palpitations and the absence of ventricular premature beats, highlighting the potential for calcium supplementation in acute cases where electrolyte imbalances affect cardiac electrophysiology. A study [17] reported the use of intravenous (IV) lidocaine as a potential therapeutic option or "bridge to transplant" for select patients with LQT3 who continue to experience arrhythmias despite standard treatments. As the prevalence of congenital LQTS increases and the risk of SCD persists, healthcare providers are likely to encounter challenging management decisions.

European Guidelines for the Management of Long QT Syndrome

The 2022 European Society of Cardiology (ESC) guidelines recommend β-blockers as the initial therapy for all LQTS patients irrespective of heart rate, though the impact on those with slow heart rates remains unclear [27]. Mexiletine is advised specifically for LQT3 patients displaying QTc prolongation and has been established as the preferred anti-arrhythmic due to its effectiveness in preventing recurrent TdP when other medications fail. The guidelines suggest that combining mexiletine with non-selective β-blockers such as nadolol or propranolol may be more beneficial than β-blocker therapy alone for high-risk LQT1 and LQT2 patients [28]. They recommend confirming the effectiveness of mexiletine by ensuring a QTc reduction of ≥40 ms via oral testing [27].

For managing VAs and preventing SCD when pharmacotherapy is inadequate, the guidelines endorse ICD for symptomatic LQTS patients already on β-blockers and genotype-specific treatments. ICDs, though effective, may provoke sympathetic activation leading to electrical storms and are generally less well-tolerated compared to drug treatments [27]. LCSD is another non-drug strategy for those with contraindications or intolerance to ICDs; however, its effectiveness and safety on a larger scale remain uncertain, and it is primarily considered an auxiliary therapy [27].

Canadian Guidelines for the Management of Long QT Syndrome

The 2023 Canadian Cardiovascular Society Clinical Practice's update on managing patients with prolonged QT intervals emphasizes β-blockade, with nadolol or propranolol preferred, for cLQTS, while cardiac rhythm devices are rarely needed. Most TdP episodes result from noncompliance with β-blockade or QT-prolonging medications. TdP management includes β-blockade, stopping QT-prolonging drugs, correcting electrolytes, increasing magnesium, using lidocaine, and occasionally transvenous pacing, while amiodarone and procainamide are contraindicated [29]. For acquired LQTS, often caused by QT-prolonging drugs or electrolyte disturbances, risks vary within drug classes; erythromycin, citalopram, and escitalopram pose higher risks, while safer alternatives include penicillins, cephalosporins, and certain attention-deficit/hyperactivity disorder stimulants. Higher TdP risk is associated with QTc exceeding 500 ms or significant drug-induced increases, particularly in women, the elderly, and those with structural heart disease. ECG monitoring is advised before starting QT-prolonging drugs, especially with electrolyte disturbances or existing QT-prolonging medications, and underlying cLQTS should be considered if QTc does not normalize after removing the causative agent [29].

Artificial Intelligence in the Diagnosis and Management of Long QT Syndrome

LQTS diagnosis and management have evolved with the understanding that mutations can lead to varying degrees of QT prolongation and arrhythmogenic risk. Advanced genetic testing and the use of artificial intelligence (AI) in ECG analysis are improving the identification of patients, even those with concealed LQTS. AI models, trained on large datasets of genetically confirmed cases, can identify subtle ECG features beyond the QT interval, enhancing diagnostic accuracy and aiding in risk stratification. These advancements are crucial as timely diagnosis and personalized management strategies can significantly reduce the risk of life-threatening arrhythmias in LQTS patients.

AI is revolutionizing the diagnosis and management of LQTS. Traditional methods of diagnosing LQTS rely heavily on the manual interpretation of ECGs to measure the QT interval, which can be subjective and sometimes insufficient, especially in cases of concealed LQTS where the QT interval appears normal. AI, particularly through the application of deep learning algorithms, has the capability to analyze vast amounts of ECG data quickly and accurately, identifying subtle patterns and features that may not be apparent to the human eye. For instance, AI models have been trained to detect LQTS by examining not just the QT interval but also additional ECG features such as the onset of the QRS complex. These advanced algorithms achieve high accuracy, often outperforming human experts, and offer a non-invasive, cost-effective method for early detection and risk stratification [30]. A study in 2021 developed an AI-ECG model that could distinguish patients with electrocardiographically concealed LQTS, offering a simple and inexpensive method for early detection [31]. A deep neural network (DNN) was trained on ECGs from over 250,000 patients, with strong agreement between the DNN-predicted values and the gold standard QTc values [30]. The DNN performed robustly when tested on a prospective genetic heart disease clinic population, relying on a two-lead ECG input, which was also effective with a mobile ECG device. The AI algorithm enhanced the detection of concealed LQTS by analyzing T-wave morphology, improving diagnostic accuracy [30].

A 2022 study trained convolutional neural network models to identify genotype-positive LQTS patients using ECGs from large datasets. These models outperformed expert cardiologists in specificity and identified new diagnostic features, such as the onset of the QRS complex, previously not associated with LQTS [32]. Another study in 2024 analyzed ECGs from the Hearts in Rhythm Organization Registry and found that the deep learning model improved the detection of congenital LQTS and differentiated between common genetic subtypes [33].

A retrospective AI-based analysis compared a fully convolutional network (FCN) with a novel model, XceptionTime, using 12-lead ECGs from genetically confirmed LQTS patients and a control cohort. The XceptionTime model outperformed the FCN, achieving a higher balanced accuracy (91.8% vs. 83.6%) and demonstrating robust predictive accuracy regardless of age and QTc parameters.

Limitations

A limitation of this study was the inability to access all the full-text papers available within the criteria outlined. Studies included in this review vary in their methodologies, such as study design, patient populations, interventions, and outcome measures, making it challenging to compare and synthesize their results.

## Conclusions

In conclusion, the management of LQTS presents complex challenges that require a multi-faceted approach. Similarly, management involves a strategic combination of pharmacological treatment, lifestyle modifications, and, in severe cases, ICD placement. Pharmacological treatments, particularly β-blockers, play a critical role, although their effectiveness and safety profiles necessitate careful consideration based on individual patient risk factors and genetic backgrounds.

Additionally, lifestyle adjustments and psychological support are indispensable components of a comprehensive treatment plan, addressing not only the physiological aspects of these conditions but also the psychological impacts, which are profound yet often under-recognized. The involvement of a multidisciplinary team, including cardiologists, genetic counselors, and psychologists, is crucial to optimize patient outcomes and improve quality of life. Despite advances in treatment, significant gaps remain in the understanding of the pathophysiology and optimal management strategies for LQTS. Future research should focus on refining risk stratification models, developing new therapeutic modalities, and generating robust data to guide treatment decisions. As our understanding evolves, so too will the strategies to mitigate the risks associated with these potentially lethal cardiac arrhythmias, aiming for a more personalized medicine approach that considers the unique genetic and clinical profile of each patient.

## Appendices

**Table 3:** Results for the analysis of the study quality or bias using the JBI critical appraisal tools for the included studies

Checklist question	Ahn et al. [12]	Krøll et al. [13]	Niaz et al. [14]	Takasugi et al. [15]	Wang et al. [16]
Were there clear criteria for inclusion in the case series?	Yes	Yes	Yes	Yes	Yes
Was the condition measured in a standard, reliable way for all participants included in the case series?	Yes	Yes	Yes	Yes	Yes
Were valid methods used for the identification of the condition for all participants included in the case series?	Yes	Not clear	Yes	Yes	Yes
Did the case series have the consecutive inclusion of participants?	Yes	Yes	Yes	Yes	Yes
Did the case series have a complete inclusion of participants?	Yes	Yes	Yes	Yes	Yes
Was there clear reporting of the demographics of the participants in the study?	Yes	Yes	Yes	Yes	Yes
Were the outcomes or follow-up results of cases clearly reported?	Yes	Yes	Yes	Yes	Yes
Was there clear reporting of the presenting sites’ or clinics’ demographic information?	Yes	Yes	Not clear	Yes	Not clear
Was the statistical analysis adequate?	Yes	Yes	Yes	Yes	Yes
